# Characterisation of RT-QuIC negative cases from the UK National CJD Research and Surveillance programme

**DOI:** 10.1007/s00415-024-12345-w

**Published:** 2024-04-10

**Authors:** Dominic Ng, Neil Watson, Eugene Ace McDermott, Hatice Kurucu, David Summers, Mary Andrews, Alison Green, Marcelo Barria, Janet McKenzie, Johnny Tam, Colin Smith, Suvankar Pal

**Affiliations:** UK National CJD Research and Surveillance Unit, Centre for Clinical Brain Sciences, University of Edinburgh, Edinburgh, UK

**Keywords:** Creutzfeldt–Jakob disease, Sporadic, Prion, CSF, RT-QuIC, Surveillance

## Abstract

**Introduction:**

Incorporation of the real-time quaking-induced conversion (RT-QuIC) assays for diagnosis of sporadic Creutzfeldt-Jakob disease (CJD) has transformed diagnosis largely related to its extremely high specificity. However, the test has a c.10% false-negative result and we aim to characterize the clinical features, investigation profile, and molecular subtype in this cohort of patients.

**Methods:**

250 individuals diagnosed with definite sporadic CJD were identified from the UK National CJD Research and Surveillance Unit from 2012 to 2023. We compared the clinical features and investigation profile in those with a negative CSF RT-QuIC to those with a positive RT-QuIC.

**Results:**

27 individuals (10.8%) were CSF RT-QuIC negative. Median age of onset was younger (62 years vs 68 years, *p* = 0.002), median disease duration was longer (4.4 months vs 10.5 months, *p* < 0.001), and these individuals were less likely to present with gait difficulties (73% vs 93%, *p* = 0.003) or motor symptoms (62% vs 80%, *p* = 0.04). The sensitivity of electroencephalography and diffusion-weighted MRI were similar in both groups. In those who were RT-QuIC negative, there was an overrepresentation of the VV1 (32% vs 1%) and MM2 molecular subtypes (21% vs 3%). Co-occurring neurodegenerative disease was found in 33% (9/27) of those who were RT-QuIC negative.

**Conclusions:**

Individuals with sporadic CJD and a negative CSF RT-QuIC present with younger age of onset, different clinical features and are over-represented with the VV1 and MM2 subtypes of sporadic CJD. Further work is required to better understand the biochemical properties contributing to RT-QuIC negative results in these cases.

**Supplementary Information:**

The online version contains supplementary material available at 10.1007/s00415-024-12345-w.

## Introduction

Sporadic Creutzfeldt–Jakob Disease (sCJD) is a rapidly progressive and fatal human prion disease characterised by cognitive, neuropsychiatric, and motor impairment. Prion diseases are characterized by the accumulation and propagation of transmissible misfolded proteins, with tissue distribution varying between disease aetiologies posing significant infection control and public health risks [1]. sCJD has a heterogeneous clinical presentation but most often presents with rapidly progressive dementia with associated myoclonus, ataxia, motor symptoms and visual symptoms [1]. The diagnosis of sporadic CJD was previously based on non-specific CSF markers of rapid neuronal injury (e.g., 14-3-3, neuron-specific enolase) alongside archetypal clinical features, electroencephalogram (EEG), and MRI. Recent incorporation of the real-time quaking-induced conversion (RT-QuIC) test in international consensus diagnosis criteria marked a significant step change in the diagnostic approach to sCJD achieving a very high specificity (98–100%) [1, 2]. Despite this, however, RT-QuIC has its limitations due to its sensitivity with approximately 10% of individuals with sCJD testing negative on RT-QuIC [1–9].

Individuals with negative CSF RT-QuIC have been identified to be younger, and with a longer disease duration with two studies also reporting reduced likelihood of developing ataxia [8–10]. This is likely partially explained by an overrepresentation of molecular subtypes for which RT-QuIC is less sensitive as defined by a combination of a polymorphism of codon 129 in the prion protein (*PRNP*) gene (methionine homozygous (MM); methionine-valine heterozygous (MV); valine-valine homozygous (VV)) and PrP^Sc^ isotype (either type 1A or type 2A) [11]. In particular, the molecular subtypes VV1 (~ 1% of those with sporadic CJD) and MM2 (~ 5% of those with sporadic CJD) are associated with lower RT-QuIC sensitivity compared with the more common molecular subtypes found in sCJD [1, 4, 8, 9]. Rhoads et al*.* identified a significantly lower sensitivity in VV1 and MM2 subtypes but other studies have only identified a lower sensitivity in the MM2 subtype or have been too limited in sample size to conduct statistical analysis given the rarity of these subtypes [4, 8–10]. Furthermore, none of these studies have included whether co-morbid neurodegenerative disease impact the RT-QuIC sensitivity. While co-morbid neuropathology has been previously been found to have no effect on the sensitivity of 14-3-3 this hypothesis in relation to RT-QuIC testing has not previously been explored to our knowledge [12].

We performed a study to compare the clinical characteristics, imaging, CSF biomarkers, and molecular subtypes in those with a positive and negative RT-QuIC in those with definite (autopsy-confirmed) sCJD identified from a national surveillance programme with a high rate of case ascertainment. Furthermore, this study will assess for the occurrence of any other neurodegenerative disease identified on autopsy that may confound RT-QuIC findings.

## Methods

### UK National CJD Surveillance

Data for this study were obtained from the UK National Creutzfeldt-Jakob Disease Research and Surveillance Unit (NCJDRSU). The unit receives referrals from healthcare professionals throughout the UK for individuals with potential CJD. Unit clinicians conduct structured detailed clinical and epidemiological evaluations recording CSF and imaging biomarkers, *PRNP* genotyping, and findings on neuropathology. The unit serves as the UK national reference laboratory for RT-QuIC testing and all referred individuals undergo neuroimaging which is reviewed by a neuroradiologist with expertise in prion disease. We reviewed data regarding cases assessed from the 1st of January 2012 to 5th September 2023. This period was chosen as this coincided with routine use of RT-QuIC by the NCJDRSU. To better assess how the demographics and clinical characteristics differed between those with definite CJD and those with probable CJD we started by comparing these two populations. The definitions for this were based on consensus international diagnostic criteria from 2010 which were subsequently updated in 2017 [13]. We excluded individuals with probable CJD who were still alive at the time of analysis.

### Clinical data

Clinical data were recorded following NCJDRSU physician assessments using a standardised questionnaire including structured information gathered from the patient, the patient’s relatives, and hospital records. This includes information on presenting symptoms, evolution of symptoms, investigation results (including EEG and MRI), genetics, and neuropathology results. Disease duration was classed as the time between the first symptom noted and death. Presenting symptoms were categorized into symptom complexes including psychiatric & behavioural disturbance, cognitive impairment, motor and gait abnormalities, visual disturbances, headache, sleep disturbance, dizziness and vertigo, fatigue and malaise, sensory disturbance, speech disturbance, language disturbance, auditory disturbance, seizures, and other (Supplementary Materials 2).

### Neuroimaging and electroencephalography findings

For each individual diffusion weighted magnetic resonance imaging (DW-MRI) was classed as either diagnostic or not diagnostic for sCJD based on the expert evaluation of a NCJDRSU consultant neuroradiologist (D.S.) with an expertise in prion disease based on international diagnostic criteria [13]. If classed as diagnostic the regions of the brain affected were then categorized by location. Our study reports on whether there was cortical involvement only, basal ganglia involvement only, or both basal ganglia and cortical involvement. EEG recordings were categorized as diagnostic if generalised periodic sharp wave complexes (PSWC) were evident.

### Cerebrospinal fluid analysis

Cerebrospinal fluid (CSF) 14-3-3 (up to April 2022) and RT-QuIC analysis was conducted at the NCJDRSU laboratory after sample transfer from referring hospitals. RT-QuIC was conducted using the first generation technique with full-length hamster rPrP only and was reported as either positive or negative [14]. 14-3-3 was classified as either positive or negative. A 14-3-3 result classed as weak positive was classed as negative.

### Neuropathological analysis

All brain material was analyzed for presence of prion pathology using 12F10 and KG9 immunohistochemistry. Routine screening was also completed for amyloid-β, tau, α-synuclein and pTDP-43 co-pathology. Biochemical classification was performed after western blotting based on the molecular weight of the abnormal prion protein and reported as either 21 kDa (Type 1A) or 19 kDa (Type 2A). Cases with both type 1A and type 2A isoforms were classed as mixed and in cases with dual isoforms we classified by the dominant isoform, with the less prominent in brackets (e.g., MM1 (+ 2)).

### Prion protein (*PRNP*) genotyping

*PRNP* gene sequencing was routinely offered to all individuals diagnosed with sCJD. All cases with a pathogenic mutation (confirming an inherited prion disease) were excluded from this study. Codon 129 polymorphism (MM, MV, VV) was evaluated with biochemical classification of the abnormal prion protein to form molecular subtypes.

### Statistical analysis

Python 3.11 was used for all statistical calculations. Categorical variables were compared using chi-squared test or Fisher’s exact test. Shapiro–Wilk Test was used to assess if data were normally distributed. Parametric continuous data were assessed with ANOVA or t-test and non-parametric data were assessed using a Mann–Whitney *U* Test. Disease duration was analyzed using the Kaplan–Meier method and Wilcoxon rank sum test.

## Results

We identified 250 individuals with definite sCJD (autopsy-confirmed) within the NCJDRSU study cohort and the demographics, investigation results, and genetic profile are summarized in Table 1. There was no missing data on sex, ethnicity, or disease duration. Information about age at diagnosis was missing for one individual, and 14-3-3 data were missing for four individuals. Codon 129 status was not available for 26 individuals, there was 1 individual with missing data on age, and 11 individuals with missing data on 14-3-3.

**Table 1:** showing the demographics and investigation results of those with definite CJD referred after 2012 to the National CJD Research and Surveillance Unit

	Result (*n* = 250)
Age of onset	
Mean ± SD	67.2 ± 9.6
Median (IQR)	68 (62–73)
Range	25–94
Sex, Male, n	127/250 (51%)
Duration (months), median (IQR)	4.5 (2.8–9.2)
Investigation results, *n*	
CSF	
RT-QuIC	
Positive	223/250 (89%)
Negative	27/250 (11%)
14-3-3	
Positive	168/239 (70%)
Negative	71/239 (30%)
MRI Brain	
Positive	195/231 (84%)
Negative	36/231 (16%)
EEG	
Positive	74/223 (33%)
Negative	149/223 (67%)
Codon 129 polymorphism	
MM	138/224 (62%)
MV	39/224 (17%)
VV	47/224 (21%)
Prion protein subtype	
Type 1	118/199 (59%)
Type 2	57/199 (29%)
Mixed/other	24/199 (12%)

RT-QuIC, real-time quaking-induced conversion; MM, Methionine homozygous; MV, Methionine-valine heterozygous; VV, Valine homozygous; n, number; IQR, interquartile range; EEG, electroencephalogram

### Comparing cohort characteristics in those with definite and probable sCJD and RT-QuIC negative results

As a decision to proceed with a post-mortem is influenced by an individual’s clinical and imaging characteristics we assessed for any differences between these two cohorts by comparing those with a post-mortem to those without a post-mortem among those with a negative RT-QuIC.

We identified 69 individuals with probable or definite sCJD and a negative RT-QuIC. Table 2 summarizes the differences in the post-mortem group (*n* = 27) compared to those without post-mortem (*n* = 42) for all individuals with a negative RT-QuIC result. This analysis demonstrated a significant difference (*p* = 0.01) in age of disease onset between those with sporadic CJD and a negative RT-QuIC result who had a post-mortem and those who did not. Those with a post-mortem were younger (median = 62 years; range = 25–79 years) compared to those with no post-mortem (median = 69 years; range = 46–86 years). However, there was no significant difference found between gender (*p* = 0.64), ethnicity (*p* = 1), disease duration (*p* = 0.06), or 14-3-3 result (*p* = 1). There was no significant difference in symptoms at diagnosis found in those with a post-mortem and those without a post-mortem and there was no significant difference (*p* = 0.65) in the sensitivity of DW-MRI.

**Table 2:** Demographics and Test comparing individuals with post-mortems to those without post-mortem in RT-QuIC negative individuals

	Post-mortem (*n* = 27)	No post-mortem (*n* = 42)	*P* value
Sex, male, *n*	13/27 (48%)	23/42 (55%)	0.64
Ethnicity, white, n*	25/27 (96%)	39/42 (93%)	1
Age, years, mean ± SD	60.70 ± 11.07	67.5 ± 10.12	**0.01**
	Median = 62	Median = 69	
	IQR = 56–68	IQR = 61–73	
Disease duration, months, mean ± SD, range, median	12.07 ± 8.82, 30.23	7.30 ± 7.24, 36.70	0.06
	Median = 10.51	Median = 4.43	
	IQR = 4.1–17.3	IQR = 2.9–9.6	
14-3-3 positive, *n*	13/27 (48%)	18/37 (49%)	1
DW-MRI positive, *n*	25/26 (96%)	28/35 (80%)	0.65
EEG Positive, *n*	6/26 (30%)	2/14 (14%)	0.51
Codon 129—MM	8/26 (31%)	10/25 (40%)	0.43
Codon 129—MV	7/26 (27%)	9/25 (36%)	
Codon 129—VV	11/26 (42%)	6/25 (24%)	

MM, Methionine homozygous; MV, Methionine-valine heterozygous; VV, Valine homozygous; DW-MRI, Diffusion weighted magnetic resonance imaging; EEG, electroencephalogram; n, number; SD, standard deviation; IQR, interquartile range; EEG, electroencephalogram

### Comparing RT-QuIC negative cases with RT-QuIC positive cases in those with definite sCJD

In the NCJDRSU cohort, we identified 250 individuals with a post-mortem between the 1st of January 2012 and 5th September 2023. The study populations demographics and CSF/Imaging results are summarized in Table 1. A comparison was then performed assessing for differences in the demographics, clinical features, and investigation results in these two cohorts (Table 3).

**Table 3:** Demographics and Test results of those with definite CJD in those with positive or negative RT-QuIC

	Negative RT-QuIC (*n* = 27)	Positive RT-QuIC (*n* = 223)	*P*-value
Sex, male, *n*	13/27 (48%)	114/223 (51%)	0.84
Ethnicity, white, n*	25/27 (93%)	212/220 (96%)	0.94
Age, years, mean ± SD	60.70 ± 11.07	68.02 ± 9.14	**0.002**
	Median: 62	Median: 68	
	IQR: 56–68	IQR: 63–73	
Disease duration, months, median (IQR)	10.51 (4.1–17.3)	4.37 (2.5–8.0)	** < 0.001**
14-3-3 sensitivity, *n*	13/27 (48%)	163/217 (75%)	**0.003**

RT-QuIC, real-time quaking-induced conversion; SD, standard deviation; IQR, interquartile range; n, number

We identified that those who were RT-QuIC negative were significantly younger (*p* = 0.002) with a median age of 62 years (IQR: 56–68; range: 25–79) compared to RT-QuIC positive individuals who had a median age of 68 years (IQR: 63–73; range: 32–94). The number of individuals with a positive 14-3-3 was also significantly lower (*p* = 0.003) in those who were RT-QuIC negative (13/27; 48%) than those who were RT-QuIC positive (163/217; 75%).

**Fig. 1:**
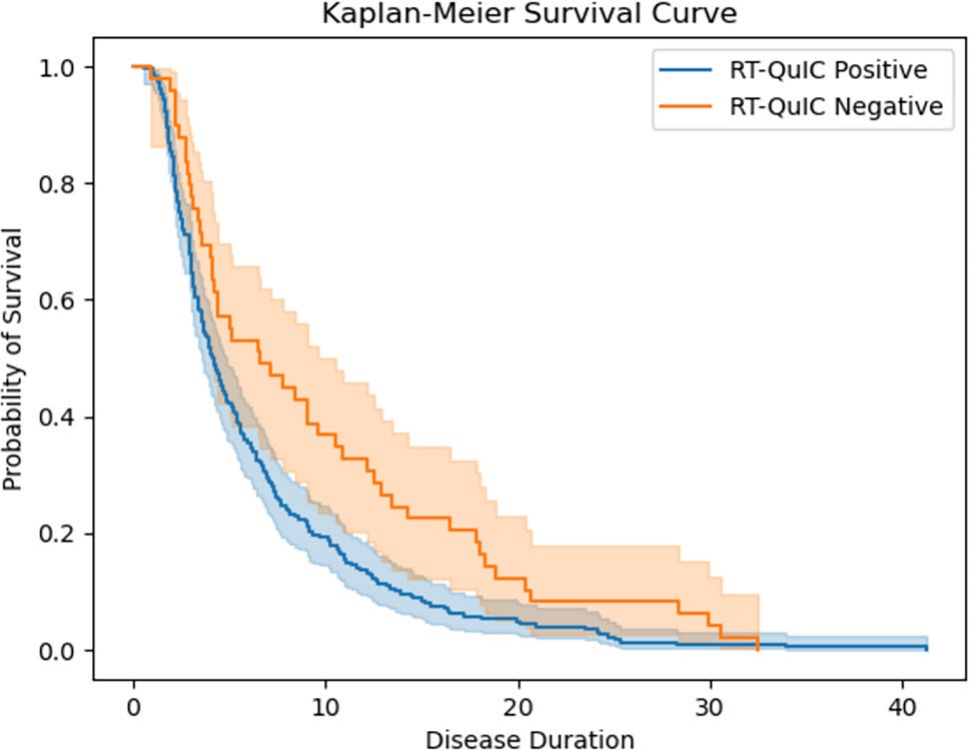
A Kaplan–Meier curve comparing duration of disease in those with a positive and negative RT-QuIC and definite sCJD

Furthermore, there was a significant difference (logrank *p* < 0.001) in disease duration (Fig. 1) with those who were RT-QuIC negative (median = 10.51 months; IQR: 4.1–17.3 range = 2.23–32.46) surviving longer than those who were RT-QuIC positive (median = 4.37 months; IQR: 2.5–8; range = 0.53–41.26).

### Comparing presenting symptoms in those with a positive and negative RT-QuIC result

**Fig. 2:**
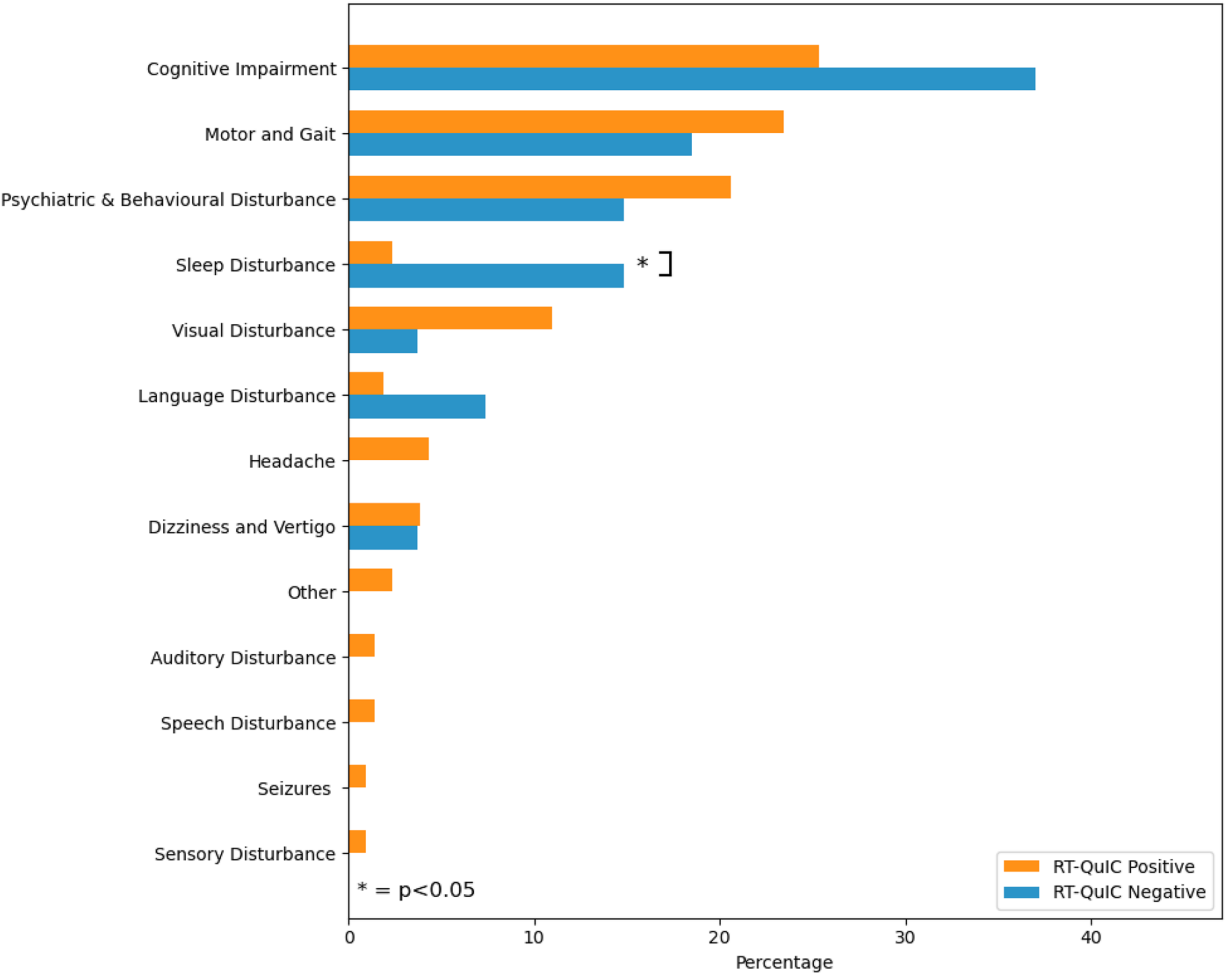
A figure comparing first symptoms in those with a positive and negative RTs-QuIC test

We compared the difference in presenting symptoms between these two cohorts (Supplementary Table 2; Fig. 2). Those who were RT-QuIC negative were significantly (*p* = 0.001) more likely to present with sleep disturbance (4/27; 15%) compared to those who were RT-QuIC positive (5/211; 2%). In these individuals, the presenting complaints were insomnia (*n* = 2), REM sleep-behaviour disorder (*n* = 1), and hypersomnia (*n* = 1). The most common initial symptoms in both cohorts were cognitive impairment and motor/gait impairment. 12 individuals (5%) (all in the RT-QuIC positive cohort) had missing information regarding their first symptom.

### Symptoms present at diagnosis

**Fig. 3:**
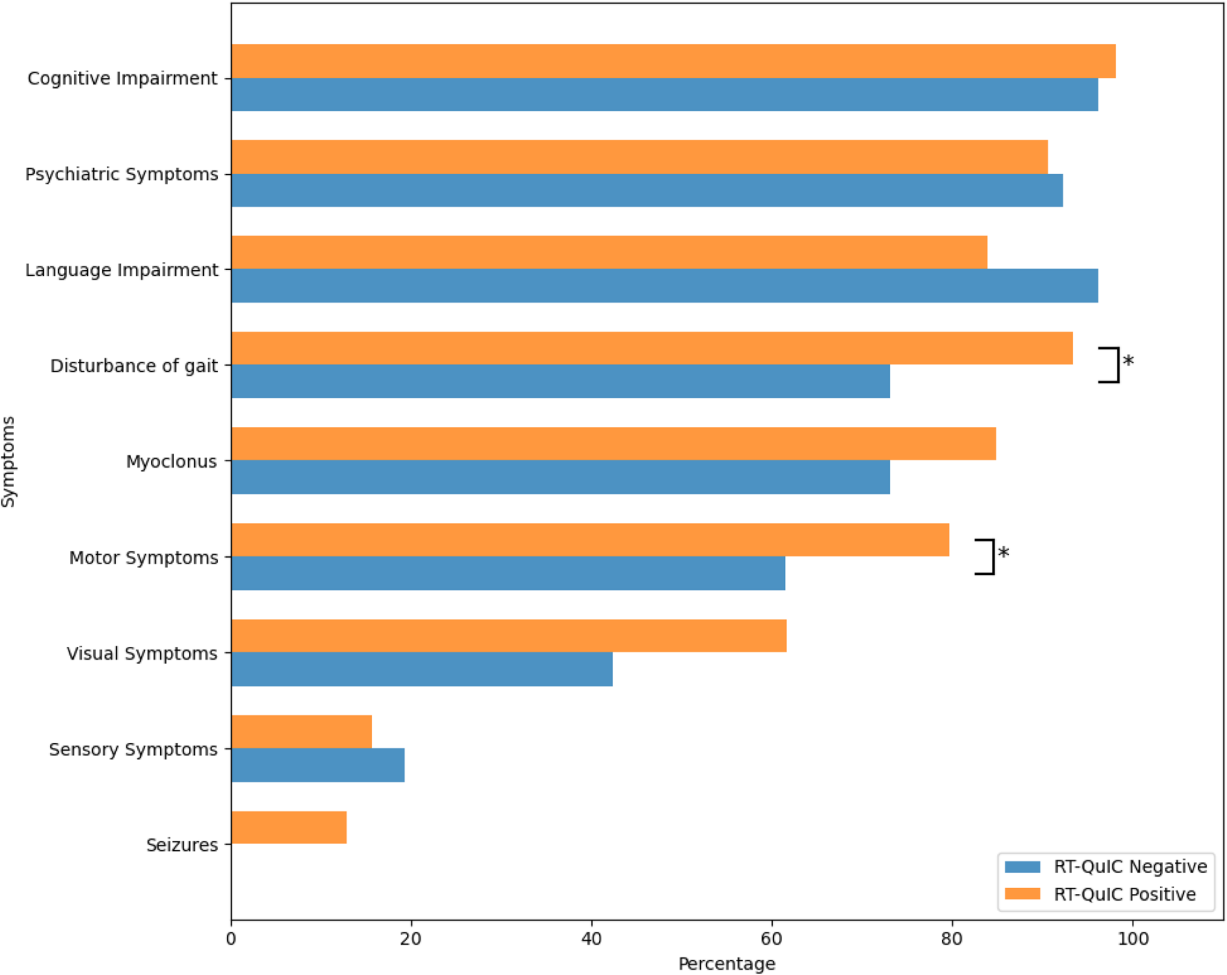
Comparing symptoms at diagnosis in those with a positive and negative RT-QuIC

**Fig. 4:**
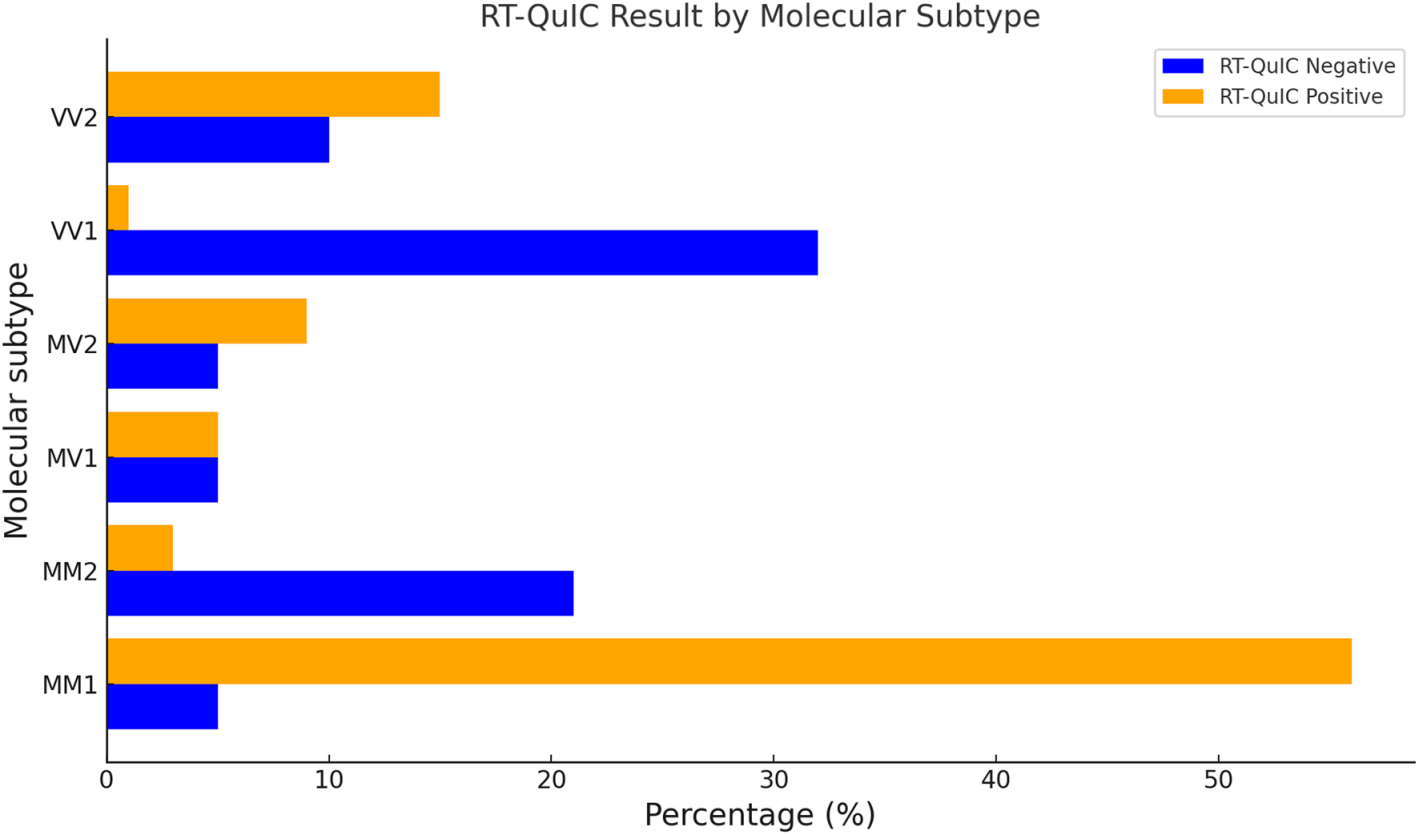
A bar chart comparing molecular subtype in those who are RT-QuIC negative and RT-QuIC positive (minus those with dual prion protein types)

Those who were RT-QuIC negative were less likely (*p* = 0.003) to have disturbance of gait (19/26; 73%) than those who were positive (197/211; 93%). RT-QuIC negative individuals were more likely (*p* = 0.04) to have motor symptoms (16/26; 62%) compared to RT-QuIC positive individuals (168/211; 80%). These results are summarized in Supplementary Table 3 and Fig. 3. There were 13 individuals (5%) with missing information on symptoms at diagnosis (12 in the RT-QuIC positive cohort and 1 in the RT-QuIC negative cohort) (Fig. 4).

### DW-MRI and EEG investigations

There was no significant difference (*p* = 0.09) between the sensitivity of DW-MRI in those who were RT-QuIC positive (83%) and those who were RT-QuIC negative (96%) (Table 4). There was also no significant difference (*p* = 0.35) found between the sensitivity of EEG in those who were RT-QuIC positive and RT-QuIC negative. In those with a diagnostic DW-MRI, there was no difference (*p* = 0.82) in the areas of the brain affected on DWI when categorised as cortical only, cortical and basal ganglia, and basal ganglia only in those who were RT-QuIC positive and RT-QuIC negative. There were 20 individuals (8%) with missing MRI results and 27 individuals (11%) with missing EEG results (Tables 4 and 5).

**Table 4:** Comparing the sensitivity of DW-MRI and EEG in those who are RT-QuIC positive and RT-QuIC negative

	RT-QuIC negative	RT-QuIC positive	*p* value
DW-MRI, sensitivity (*n*)	25/26 (96%)	173/204 (85%)	0.09
DWI—cortical only, *n*	6 (24%)	33 (20%)	0.35
DWI—cortical and basal, *n* ganglia, *n*	17 (68%)	114 (67%)	
DWI—Basal ganglia only, *n*	2 (8%)	26 (15%)	
Diagnostic EEG, *n*	6/26 (30%)	68/197 (34%)	0.28

RT-QuIC, real-time quaking-induced conversion; n, number; DW-MRI, Diffusion weighted magnetic resonance imaging; EEG, electroencephalogram

**Table 5:** Comparing codon 129 polymorphism in those with a positive and negative RT-QuIC

Codon 129	RT-QuIC negative	RT-QuIC positive	*p*-value
MM, *n*	8/26 (31%)	130/198 (66%)	0.002
MV, *n*	7/26 (27%)	32/198 (16%)	
VV, *n*	11/26 (42%)	36/198 (18%)	

RT-QuIC, real-time quaking-induced conversion; MM, Methionine homozygous; MV, Methionine-valine heterozygous; VV, Valine homozygous; n, number

### PRNP codon 129 status

There was a significant difference (*p* = 0.002) identified between groups (Table 5; Supplementary Fig. 1). The MM genotype was present in 27% (8/26) of RT-QuIC negative individuals compared to 58% (130/198) of RT-QuIC positive individuals (standardised residual of 2). The VV genotype was present in 42% (11/26) of RT-QuIC negative individuals compared to 16% (36/198) of RT-QuIC positive individuals (standardised residual of 2.3). There was missing codon 129 status for 26 individuals (10%) of which 25 were in the RT-QuIC positive cohort.

**Table 6:** A table comparing molecular subtype in those who are RT-QuIC negative and RT-QuIC positive

Molecular subtype	RT-QuIC negative	RT-QuIC positive
MM1, *n*	1/19 (5%)	102/181 (56%)
MM2, *n*	4/19 (21%)	6/181 (3%)
MV1, *n*	1/19 (5%)	9/181 (5%)
MV2, *n*	1/19 (5%)	17/181 (9%)
VV1, *n*	6/19 (32%)	1/181 (1%)
VV2, *n*	2/19 (11%)	27/181(15%)
MM1(+ 2), *n*	2/19 (11%)	9/181 (5%)
MM2(+ 1), *n*	0/19 (0%)	0/181 (0%)
MV1(+ 2), *n*	0/19 (0%)	2/181 (1%)
MV2(+ 1), *n*	1/19 (5%)	1/181 (1%)
VV2(+ 1), *n*	0/19 (0%)	1/181 (1%)
MM1 LMWt, *n*	0/19 (0%)	1/181 (1%)
VV1 (+ 2), *n*	0/19 (0%)	1/181 (1%)
MV Intermediate*, *n*	1/19 (5%)	0/181 (0%)
MV2K + C, *n*	0/19 (0%)	1/181 (1%)

RT-QuIC, real-time quaking-induced conversion; MM, Methionine homozygous; MV, Methionine-valine heterozygous; VV, Valine homozygous; n, number

^*^Intermediate was a PrP isotype with a molecular weight of 20 kDa

### Gene sequencing and mutations

There were no known disease-causing mutations identified although one had a *PRNP* A117a silent mutation. *PRNP* gene sequencing was available in 17/27 (63%) of individuals with definite CJD who were RT-QuIC negative. None of the cases without sequencing had a family history of neurodegenerative disease.

### Molecular subtypes in those who are RT-QuIC negative

There was significant over-representation of the VV1 and MM2 subtypes in those who were RT-QuIC negative (Table 6; Fig. 4). The VV1 subtype made up 32% (6/19) of those who were RT-QuIC negative while only making up 1% (1/181) of those who are RT-QuIC positive. The MM2 subtype makes up a disproportionately high number in those who are RT-QuIC negative (4/19; 21%) compared to those who are RT-QuIC positive (6/181; 3%). Furthermore, the MM1 subtype has a high representation in those who are RT-QuIC positive (102/181; 56%) while it makes up little of those who are RT-QuIC negative (1/19; 5%). There were 50 individuals with missing molecular subtype (8 in the RT-QuIC negative cohort and 42 in the RT-QuIC positive cohort).

### Neuropathological profiles

There was co-occurrence of severe pathology in 9/27 (33%) of the individuals with definite CJD who tested negative for RT-QuIC (Supplementary Table 4). There were 5 (19%) individuals with co-occurrence of severe amyloid beta pathology. There were 4 (15%) with severe tau pathology (3 in those with severe amyloid plaques; 1 in those without amyloid plaque deposition). In this case it was noted by two pathologists that the tau deposition and neurofibrillary tangles were out of keeping with normal prion protein deposition. One individual was noted to have severe and widespread Lewy body disease. There was evidence of Lewy body inclusions on alpha synuclein staining in the substantia nigra and locus coeruleus with thickened Lewy neurites within these nuclei. There were further Lewy body inclusions in the parietal and temporal cortex as well as basal ganglia and thalamus. There were two individuals noted to have severe cerebral amyloid angiopathy (CAA) of which one also had severe Ab and tau pathology. There was 1 patient with transactive response DNA binding protein 43 (TDP43) threads in the parahippocampal and fusiform gyri with the presence of severe arteriosclerosis. One individual had a large infarct in the right parietal lobe (3 × 2x1 cm) within the territory of the right middle cerebral artery but there was no further neurodegenerative disease identified on routine staining.

## Discussion

RT-QuIC testing, with its extremely high specificity, has transformed the diagnosis of sporadic CJD and has allowed for more accurate in-life diagnosis. Results from our study demonstrate that a small yet significant portion (~ 10%) of those with sporadic CJD have a false-negative RT-QuIC result when diagnosis is confirmed at autopsy. It is important for clinicians to understand the clinical characteristics and performance of other investigation results in this patient population. Furthermore, this study aimed to explore the contribution of underlying molecular subtypes on performance of RT-QuIC in this population and confounders relating to co-pathologies identified at autopsy.

This study identified that individuals with a negative RT-QuIC were likely to be younger at diagnosis and have a longer disease duration compared to those with a positive RT-QuIC. This finding confirms previous reports [8–10]. Of these three previous studies, only Jones et al. specifically noted the median age (RT-QuIC negative, 52; RT-QuIC positive, 66) and duration of disease (RT-QuIC negative, 710 days (~ 23.7 months); RT-QuIC positive, 147.5 days (~ 4.9 months)). Our study identifies a more modest difference between the median age of onset (RT-QuIC negative, 62 years; RT-QuIC positive, 68 years) and duration of disease (RT-QuIC negative, 10.5 months; RT-QuIC positive, 4.4 months). A reason for this variation may be related to smaller sample size (*n* = 13) and study design as Jones et al. included both those with definite and probable sCJD.

Our study also found those with a negative RT-QuIC were more likely to note sleeping difficulties as an initial symptom and at initial diagnosis were less likely to have motor symptoms and difficulties with gait. Foutz et al*.*, in their study on the sensitivity/specificity of RT-QuIC, reported on symptoms in those with a negative RT-QuIC identifying that that they were significantly less likely to present with ataxia [9]. Jones et al*.* also identified that in those with RT-QuIC negative ataxia was less frequent (31%) compared to positive patients (61%), however, this difference was not found to be significant (*p* = 0.07) [10]. RT-QuIC negative individuals in our cohort were less likely to have difficulties with gait (73% vs 93%) or present with motor symptoms (62% vs 80%) at diagnosis. However, due to performing multiple tests of comparisons our result regarding reduced motor symptoms (*p* = 0.04) should be interpreted with caution as it only just exceeds our threshold for significance.

Demographics and clinical features of RT-QuIC individuals are likely explained to some extent by the overrepresentation of certain underlying molecular subtypes and reflective of the characteristics of their individual clinico-pathological phenotype [11]. Those with the VV1 subtype of sporadic CJD have a younger age of onset (39–44 years) with a prolonged disease duration (15–21 months) [11, 15]. Although in depth clinical phenotyping of this sub-group is challenging due to its rarity (~ 1% of those with sporadic CJD), these individuals most commonly present with a slowly progressive dementia with psychiatric changes with a lower degree of neurological signs and symptoms on first examination [11, 15]. The low presence of gait abnormalities/motor symptoms when first diagnosed may simply reflect the earlier stage they are in their disease process. Our findings are comparable to these studies identifying that in our cohort of RT-QuIC negative individuals with definite CJD that those with the VV1 subtype had a median duration of 17.28 with a median age of onset of 53. The MM2C subtype also presents with a longer disease duration as a progressive dementia with a slower progression of neurological symptoms. Interestingly whilst our study also identified that those with the MM2 had a longer median duration of 30.24 months they also presented with a younger age of onset of 57 years which is not typical of this subtype [16, 17]. This finding is likely explained by our small sample size (*n* = 4) and larger studies are needed to assess its significance. Overall, it is likely that the presence of these subtypes and their associated clinical features explain why those with a negative RT-QuIC are more likely to present with a younger age of onset and longer duration of disease.

In our cohort, individuals with negative RT-QuIC were more likely to describe sleep difficulties as an initial symptom compared to those with a positive RT-QuIC. Of these four, only two had molecular subtyping (MM2 and MV1) with the other two only possessing codon 129 genotyping (VV and MV). None of our cohort had the MM2T subtype of CJD (sporadic fatal insomnia). Interestingly two of the four with sleeping difficulties also had significant co-pathology in the form of severe amyloid beta deposition (both Thal V) and neurofibrillary tangles corresponding to Braak & Braak IV and V. However, due to the small sample size (*n* = 4) it is difficult to draw conclusions regarding its significance.

### Performance of CSF 14-3-3, DW-MRI, and EEG

Our study is in line with previous studies that demonstrate that there is no significant difference between the sensitivity of MRI or EEG in those who are RT-QuIC negative and positive but that 14-3-3 demonstrates a lower sensitivity in RT-QuIC negative cases [9, 10]. The sensitivity of MRI was found to be high in those with definite CJD with a negative RT-QuIC (96%) with no significant difference found when comparing with those who were RT-QuIC positive. There was no difference demonstrated between the areas of diffusion restriction on DW-MRI between those who were RT-QuIC positive or negative. Due to the overrepresentation of these two subtypes (MM2 and VV1) it may be expected that this cohort would be more likely to have cortical ribboning on imaging. This is because several case series have identified that in both of these subtypes they are more likely to present with cortical ribboning with less frequent involvement of the basal ganglia [10, 15–18]. Our study findings indicate that MRI is highly sensitive in sCJD irrespective of RT-QuIC outcome. This should support clinicians tasked with making difficult decisions around palliative management in sCJD as well as public health-related actions.

EEG was found to have a low sensitivity (30%) which points to the lack of clinical utility of this test in aiding the diagnosis of sCJD in those who are RT-QuIC negative. This is slightly higher than a previous study comparing RT-QuIC negative and positive cases (16%) [10]. Furthermore, the VV1 and MM2C subtype are associated with lower sensitivity of EEG and clinicians would be advised to interpret a non-diagnostic result with caution [15, 16]. One hypothesis may be that this is due to the longer duration associated with these subtypes and this represents EEG likely finding less utility in those earlier in the disease process. Another is that these subtypes themselves are simply less likely to show periodic sharp wave complexes and further studies should attempt to explore these two possible hypotheses.

Our study identified that 14-3-3 has reduced sensitivity in those who are RT-QuIC negative, in concordance with findings of previous studies [8, 9]. This is likely secondary to a combination of reasons. The 14-3-3 protein family is highly expressed in the brain and, while raised in CJD, are a non-specific marker of neuronal damage and can be raised in strokes, seizures, paraneoplastic syndromes, and autoimmune encephalitis [19, 20]. As 14-3-3 is a marker of neuronal damage, it has been shown to have increased sensitivity the later in the disease course that the CSF is sampled [21]. Furthermore, 14-3-3 has been demonstrated to have reduced sensitivity in the MM2C and VV1 subtypes and this is another possible explanation for these results [11, 13, 14]. This is supported by our findings that only 0/4 of those with MM2C and 2/6 of those with VV1 had a positive 14-3-3.

### Molecular subtype and neuropathology

Results from our study are in line with those of previous studies identifying that those with the MM2 and VV1 subtypes are over-represented in those with a negative RT-QuIC [1, 8, 9]. It is currently unclear why these molecular subtypes are unlikely to test positive on RT-QuIC. It is unlikely that longer disease duration, and possible reduced protein seeding from earlier sampling, are the cause as studies have identified that RT-QuIC is unaffected by timing of CSF collection [14]. Furthermore, RT-QuIC can even be positive in individuals with prion disease prior to symptom onset [22]. A more likely answer is that some underlying characteristic of the prion protein in those with the VV1 and MM2 subtypes causes reduced protein seeding and thus reduced fluorescence on RT-QuIC testing. Studies evaluating the effect of different CJD subtypes on RT-QuIC positivity have identified an increased lag time on VV1 testing and in the study by Peden et al*.* increased lag time on VV1 and MM2C testing [23, 24]. A study by Poleggi et al*.* reviewing the effect of different recombinant prion protein substrates on VV1 and MM2C identified similar findings. Interestingly they also found that there was increased sensitivity in the improved QuIC (IQ-CSF) compared to the prior protocol QuIC (PQ-CSF) in those with MM2C (70% vs 40%) but this was found not to be the case in those with VV1 [25, 26]. The reduced PrP^sc^ seeding efficiency in these subtypes may correlate with the atypically long durations when compared to the other variants of sporadic CJD with a median duration of 3–5 months.

### Strengths and weaknesses of the study

The UK NCJDRSU surveillance cohort, as a centre for national referrals, has a high rate of case ascertainment as well as dedicated clinical phenotyping via structured clinical assessments (completed by a trained clinician). This study is also the first to directly compare the clinical, imaging, genetic, and biochemical characteristics in RT-QuIC negative and positive individuals in those with definite CJD. By doing this we aim to help clinicians when interpreting a negative RT-QuIC result and we help further characterise the clinical, imaging, and genetic profile of this cohort. All RT-QuIC test results used in this study have been performed at a single lab with a structured methodology. All imaging interpretation was performed by a neuroradiologist with expertise in prion imaging (D.S.) who is blinded to clinical parameters and reviewed in a standardised format to improve reliability and repeatability. Imaging interpretation was reported by a single individual reducing any variation secondary to inter-rater reliability.

There are some limitations to our study. We identified that those with a negative RT-QuIC and a post-mortem were likely to be younger. Furthermore, while unable to be assessed, it is likely that those with a post-mortem were more likely to have atypical disease progression/symptom profile resulting in an over-representation of those with rare molecular subtypes and those with neurodegenerative co-pathology. However, this is a broader issue affecting any study performed on a post-mortem cohort and there is no reason to suspect that our cohort is different than other studies performed on this population. There was incomplete genetic testing performed (37% of RT-QuIC negative individuals missing data on mutations) which raises the possibility that these individuals may have had genetic CJD. While we noted a high proportion of co-occurring neurodegenerative pathology in those who are RT-QuIC negative we have not compared this to a control group. Further studies should aim to assess if this significantly higher by making a comparison to those with a positive RT-QuIC result. Furthermore, our study utilized multiple tests of comparison which increases the risk of type 1 error. This is especially important when interpreting our results which just fall under our significance level such as finding reduced motor symptoms (*p* = 0.04) in our RT-QuIC negative cohort. Lastly, our study only utilized first generation RT-QuIC testing and future studies should further characterize the sensitivity of different substrates especially in the context of rare molecular subtypes of CJD.

## Conclusions

In conclusion, our study has identified that individuals with sCJD who are RT-QuIC negative are more likely to be younger with longer disease duration. They are more likely to present with sleep disturbances and on initial assessment less likely to have motor symptoms or disturbance with gait. Within this cohort there appears to be an over-representation of the VV1 and MM2 molecular subtype. However, those with post-mortems demonstrate different demographics compared to those with probable CJD introducing a degree of selection bias.

### Supplementary Information

Below is the link to the electronic supplementary material.

**Supplementary file 1:** (DOCX 56 KB)

**Supplementary file 2:** (DOCX 14 KB)

**Supplementary file 3:** (DOCX 15 KB)

**Supplementary file 4:** (DOCX 14 KB)

**Supplementary file 5:** (DOCX 20 KB)
