# Supplementary material for: Characterisation of RT-QuIC negative cases from the UK National CJD Research and Surveillance programme
Source: J Neurol. 2024 Apr 10;271(7):4216–26. doi: 10.1007/s00415-024-12345-w (PMC11233280; doi:10.1007/s00415-024-12345-w)

**Supplementary Figure 1 comparing codon 129 polymorphism in those with a positive and negative RT-QuIC result**


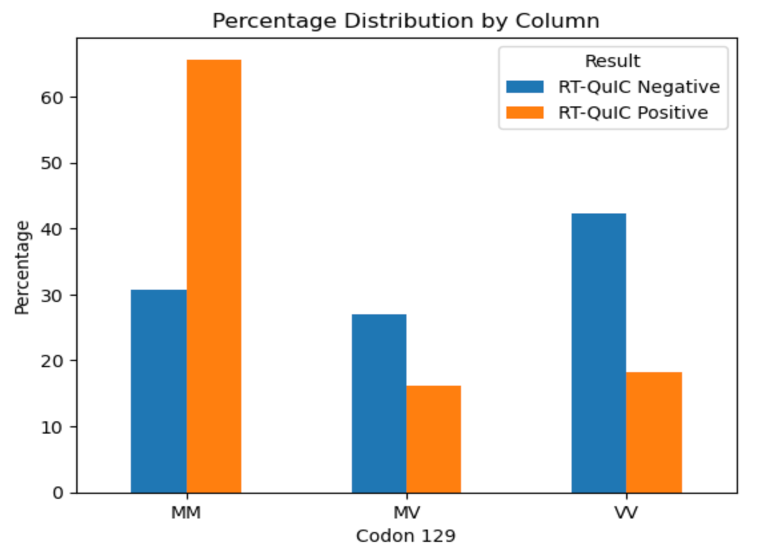

Supplement: Supplementary file 1 — (DOCX 56 KB) [file 415_2024_12345_MOESM1_ESM.docx]
