# Supplementary material for: Characterisation of RT-QuIC negative cases from the UK National CJD Research and Surveillance programme
Source: J Neurol. 2024 Apr 10;271(7):4216–26. doi: 10.1007/s00415-024-12345-w (PMC11233280; doi:10.1007/s00415-024-12345-w)
Supplement: Supplementary file 2 — (DOCX 14 KB) [file 415_2024_12345_MOESM2_ESM.docx]

**Supplementary Table 1**

| **Presenting Symptom Category** | **Symptoms** |
| --- | --- |
| Psychiatric and Behavioural Disturbance | depression, anxiety, paranoia, apathy,  withdrawal, visual and auditory  hallucinations, delusions, personality and  behavioural changes |
| Cognitive Impairment | disorientation, attention deficit, memory  impairment, navigational difficulties,  dyscalculia |
| Motor and Gait Abnormalities | unsteadiness, clumsiness, incoordination,  slowed movements, involuntary  movements, tremors, myoclonus |
| Speech Disturbance | dysarthria, dysphonia, mutism |
| Language Disturbance | dysphasia, word finding difficulties,  dyslexia |
| Visual Disturbance | diplopia, visual blurring, visual  impairment, cortical blindness, visual  illusions |
| Sensory Disturbance | numbness, tingling, pain (other than  headache) |
| Auditory Disturbance | tinnitus, hearing difficulties |
| Headache | tension-type headache, migrainous  headache |
| Sleep Disturbance | insomnia, parasomnia, REM sleep  disorder |
| Dizziness and Vertigo | vertigo, light-headedness, dizziness |
| Fatigue and Malaria | - |
| Seizures | - |
| Other | sinus congestion, coughing |
