# Supplementary material for: Characterisation of RT-QuIC negative cases from the UK National CJD Research and Surveillance programme
Source: J Neurol. 2024 Apr 10;271(7):4216–26. doi: 10.1007/s00415-024-12345-w (PMC11233280; doi:10.1007/s00415-024-12345-w)
Supplement: Supplementary file 3 — (DOCX 15 KB) [file 415_2024_12345_MOESM3_ESM.docx]

**Supplementary Table 2: Comparing first symptom noted in those with a positive and negative RT-QuIC**

|  | Negative RT-QuIC | Positive RT-QuIC | p value |
| --- | --- | --- | --- |
| Cognitive Impairment, n | 10/27 (37%) | 53/211 (25%) | 0.25 |
| Motor and gait, n | 5/27 (19%) | 49/211 (23%) | 0.81 |
| Psychiatric and  Behavioural disturbance, n | 4/27 (15%) | 43/211 (20%) | 0.61 |
| Sleep Disturbance, n | 4/27 (15%) | 5/211 (2%) | **0.001** |
| Visual Disturbance, n | 1/27 (4%) | 23/211 (11%) | 0.33 |
| Language Disturbance, n | 2/27 (7%) | 4/211 (2%) | 0.14 |
| Headache, n | - | 9/211 (4%) | 0.6 |
| Dizziness and Vertigo, n | 1/27 (4%) | 8/211 (4%) | 1 |
| Other, n | - | 5/211 (2%) | 1 |
| Auditory Disturbance, n | - | 3/211 (1%) | 1 |
| Speech Disturbance, n | - | 3/211 (1%) | 1 |
| Seizures, n | - | 2/211 (1%) | 1 |
| Sensory Disturbance, n | - | 2/211 (1%) | 1 |

**Abbreviations:** RT-QuIC, real-time quaking-induced conversion; n, number
