# Supplementary material for: Characterisation of RT-QuIC negative cases from the UK National CJD Research and Surveillance programme
Source: J Neurol. 2024 Apr 10;271(7):4216–26. doi: 10.1007/s00415-024-12345-w (PMC11233280; doi:10.1007/s00415-024-12345-w)
Supplement: Supplementary file 4 — (DOCX 14 KB) [file 415_2024_12345_MOESM4_ESM.docx]

**Supplementary Table 3: Comparing symptoms at diagnosis in those with a positive and negative RT-QuIC**

|  | Negative RT-QuIC | Positive RT-QuIC | p value |
| --- | --- | --- | --- |
| Myoclonus, n | 19/26 (73%) | 179/211 (85%) | 0.16 |
| Seizures, n | 0/26 (0%) | 27/211 (13%) | 0.05 |
| Cognitive Impairment, n | 25/26 (96%) | 207/211 (98%) | 0.44 |
| Psychiatric Symptoms, n | 24/26 (92%) | 191/211 (91%) | 1 |
| Motor Symptoms, n | 16/26 (62%) | 168/211 (80%) | **0.04** |
| Language Impairment, n | 25/26 (96%) | 181/211 (86%) | 0.24 |
| Visual Symptoms, n | 11/26 (42%) | 130/211 (62%) | 0.08 |
| Sensory Symptoms, n | 5/26 (19%) | 33/211 (16%) | 0.58 |
| Disturbance of gait, n | 19/26 (73%) | 197/211 (93%) | **0.003** |

**Abbreviations:** RT-QuIC, real-time quaking-induced conversion; n, number
