# Supplementary material for: Characterisation of RT-QuIC negative cases from the UK National CJD Research and Surveillance programme
Source: J Neurol. 2024 Apr 10;271(7):4216–26. doi: 10.1007/s00415-024-12345-w (PMC11233280; doi:10.1007/s00415-024-12345-w)
Supplement: Supplementary file 5 — (DOCX 20 KB) [file 415_2024_12345_MOESM5_ESM.docx]

**Supplementary Table 4: Describing the co-occurrence of neuropathology in those with RT-QuIC negative CJD**

| Patient | Age | Molecular Sub-Type | AB Pathology | Tau Pathology | a-syn pathology | TDP43 Pathology | Arteriosclerosis | CAA |
| --- | --- | --- | --- | --- | --- | --- | --- | --- |
| **1** | 62 | MM | - | Mild | - | None | - | - |
| **2** | 70 | VV (?) | **Moderate/Severe** | Mild (with PrP) | None | None | None | None |
| **3** | 63 | MM1 | Mild | None | None | None | None | None |
| **6** | 57 | MV2(+1) | - | - | None | - | Moderate | - |
| **7** | 71 | VV2 | Mild | Moderate (with PrP) | None | None | None | None |
| **8** | 61 | MM2C | None | None | None | None | Mild | None |
| **9** | 52 | VV1 | Mild | None | None | - | Moderate | - |
| **10** | 62 | VV1 | Mild | Mild | None | None | Moderate | **Severe** |
| **11** | 58 | MV2 | Mild | None | None | - | Moderate | None |
| **12** | 67 | - | - | - | - | - | - | - |
| **13** | 65 | MV1 | **Severe** | **Severe** | None | None | Moderate | **Severe** |
| **14** | 69 | MV (?) | Mild | None | None | None | None | None |
| **15** | 68 | VV (?) | None | Mild | Mild | **Mesial Temporal Lobe??** | **Severe** | - |
| **16** | 45 | MM1(+2) | None | None | None | - | Moderate | - |
| **17** | 61 | MV (?) | Mild | Mild | None | None | 3x2x1 infarct in right parietal | Mild |
| **18** | 50 | VV1 | Mild | None | None | None | None | None |
| **19** | 63 | VV (?) | **Severe** | Mild | None | None | Mild | None |
| **20** | 26 | VV1 | None | Mild | None | None | Mild | None |
| **21** | 51 | MM2 | Mild | None | None | None | None | None |
| **22** | 79 | VV2 | Mild | Mild | None | None | None | None |
| **23** | 61 | MV(intermediate)* | **Severe** | **Severe** | None | None | None | None |
| **24** | 60 | VV1 | None | Mild with PrP | **Severe** | None | None | None |
| **25** | 73 | MV (?) | **Severe** | **Severe** | None | None | **Moderate/Sever** | Moderate |
| **26** | 78 | MM1+2 | None | **Moderate/Severe not with PrP** | None | None | None | Mild |
| **27** | 73 | MM2 | None | None | None | None | None | None |

*Atypical PrPres fragment (20kDa) which is intermediate in size between Type 1(21kDa) and Type 2 (19kDa)
Abbreviations: MM, Methionine homozygous; MV, Methionine-valine heterozygous; VV, Valine homozygous; AB, Amyloid beta; a-syn, alpha synuclein; TDP-43, Transactive response DNA binding protein 43; CAA, cerebral amyloid angiopathy
